# Clinical Urgency of After-Hours Presentations of Dogs and Cats to a Private Small-Animal Practice in Germany: A Retrospective Study of 4579 Visits

**DOI:** 10.3390/vetsci13090899

**Published:** 2026-09-02

**Authors:** Leoni Wizenty, Marcus G. Doherr

**Affiliations:** Institute of Veterinary Epidemiology and Biostatistics, School of Veterinary Medicine, Freie Universität Berlin, 14163 Berlin, Germany; marcus.doherr@fu-berlin.de

**Keywords:** veterinary emergency, after-hours care, emergency presentations, small-animal practice, triage, medical records, canine, feline

## Abstract

This descriptive study aimed to assess the urgency of after-hours emergency visits in a small-animal practice using a structured triage system, and to characterize the associated presenting conditions and treatments. Medical records of 3189 dog and 1390 cat visits presented between 1 January and 31 December 2021 to an advanced small-animal practice were retrospectively reviewed. Retrieved data included temporal characteristics of presentation, species, signalment, clinical diagnosis, diagnostics, and treatments. Visits were retrospectively categorized using a triage approach based on the five-point Manchester Triage System (MTS). More than half of all events were classified as non-emergency visits (blue and green triage); the proportion of red triage (immediate) cases was higher among feline than canine emergency presentations. The most frequent patient presentations to the after-hours emergency service occurred in spring and summer, with May and April being the busiest; holidays and Sundays had the highest average number of cases per day during the day shift. Causes of emergency visits were mainly due to gastrointestinal disorders in dogs (32.3%) as well as in cats (28.6%). Symptomatic therapy was the most common intervention in dogs (75.1%) and in cats (49.6%), followed by diagnostics (dogs 17.5%, cats 36.0%). These findings highlight the broad spectrum of after-hours emergency presentations in small-animal practice and the high proportion of non-emergency visits, and may inform future resource allocation and emergency service planning. However, as this was a retrospective single-center study, transfer of results to other settings must be done with caution.

## Simple Summary

After-hours veterinary services provide care for animals that need medical attention when regular veterinary practices are closed. However, little is known about the types and urgency of cases presented to these services in Germany. This study examined 4579 visits by dogs and cats to a small-animal practice in Germany during 2021. Each visit was retrospectively assessed based on how urgently veterinary care was needed. More than half of the visits were classified as non-emergencies, meaning that they were considered suitable for management during regular veterinary consultation hours. Dogs were presented more often than cats, and gastrointestinal problems were the most common reason for visits in both species. Cats were more often presented with immediately life-threatening conditions than dogs. The number of visits was highest in spring and summer, particularly in April and May, and was also higher on holidays and weekends during the day. Most visits involved treatment to relieve clinical signs, while diagnostic testing and hospital care were also frequently recorded. The findings show the wide range of cases managed by after-hours veterinary services and the high proportion of non-emergency visits. They can help veterinary practices plan staffing and resources and provide better guidance to owners for when after-hours veterinary care is needed.

## 1. Introduction

After-hours veterinary emergency services in Germany are facing increasing personnel shortages and a growing workload, challenges also reported in many other countries, and mirroring developments observed in human emergency care [1,2,3,4,5,6]. Rising caseloads and limited workforce capacity have placed substantial strain on after-hours services, necessitating structured systems to ensure continued access to urgent veterinary care [7,8,9]. Therefore, after-hours veterinary emergency care in Germany is subject to national regulations, overseen by the state veterinary chambers, and is provided during legally defined periods, including weekday nights, weekends, and public holidays, with mandatory additional charges under the national Fee Schedule for Veterinarians (Gebührenordnung für Tierärzte, GOT), including a fixed emergency fee of EUR 50.00 (net) and higher fee multipliers than during regular business hours [10]. After-hours veterinary emergency service is commonly delivered by general practices participating in legally regulated regional after-hours emergency service networks, which face increasing challenges such as staff shortages, high workloads, and economic pressures [9,11]. Germany is currently facing a small-animal emergency care crisis, with long travel distances and long waiting times for owners, demonstrating the impact of veterinarian shortages on animal care [12,13,14,15]. Over recent years, the number of veterinary hospitals has declined across most German federal states, as maintaining hospital status, which requires continuous 24 h emergency service, has become increasingly demanding amid the current pressures on veterinary emergency care [9]. With no new veterinary hospitals established nationwide in 2021, after-hours emergency care capacity has been further reduced [9].

At the same time, veterinary emergency services are increasingly confronted with cases that could have been managed during regular office hours [16,17,18]. This growing proportion of non-urgent presentations, also described as “non-emergencies” or “pseudo-emergencies”, may contribute substantially to after-hours service overload and has been described as a relevant challenge in human emergency medicine worldwide [4,5,18,19,20,21,22,23,24,25,26,27,28,29].

Despite the growing importance of veterinary emergency caseload management, especially amid increasing workload and staffing challenges in after-hours services, comparable data on the types, frequencies, and urgencies of cases in small-animal practices remain very limited. This study addresses this lack of data and aims to analyze the medical records of dogs and cats presenting after hours to a small-animal veterinary practice over one year to distinguish and quantify true emergencies and non-emergency presentations. The underlying objectives of the study were: (1) to evaluate the caseload of dogs and cats presenting after hours, with respect to temporal distribution and patient characteristics; (2) to describe and systematically categorize after-hours visits according to their reasons for presentation; (3) to describe and systematically categorize the medical management and treatments of after-hours visits; and (4) to assess medical urgency of after-hours visits using a structured triage system to distinguish true emergencies from non-emergencies.

## 2. Materials and Methods

### 2.1. Study Background

The medical records analyzed in this study originate from cases presented to the emergency department of a modern, full-service advanced small-animal veterinary general practice with emergency care open 24/7, located in Berlin, Germany. The clinic team comprises ~45 employees, including around 20 veterinary professionals who actively participate in the after-hours emergency service, as well as veterinary assistants, laboratory technicians, and trainees. This structure enables a broad spectrum of diagnostic and therapeutic interventions, including inpatient facilities, outpatient infusion therapy, surgeries, and other procedures under general anesthesia, and critical care modalities such as oxygen therapy and intensive monitoring. Diagnostic capabilities are extensive and encompass complete blood analysis, fecal examinations, ultrasonography, conventional and dental radiography, and endoscopic procedures.

According to the GOT [10], after-hours emergency service periods are defined as night-time hours from 6 p.m. to 8 a.m. the following day, weekends from Friday 6 p.m. to Monday 8 a.m., and public holidays. Evening or weekend consultations provided during a veterinary practice’s regular opening hours (e.g., until 7 or 8 p.m.) are not considered after-hours emergency care under the GOT, even if these hours fall within the general time periods specified for emergency service [10]. In after-hours emergency service, veterinary practices frequently operate under reduced staffing levels and limited resources. In this study, only cases presented during the legally defined after-hours periods, taking into account the regular opening hours of the study practice, were analyzed, highlighting the challenges of providing care under limited personnel and infrastructure. This approach ensured that the study specifically addressed patterns of emergency service utilization rather than the broader population of all emergencies presenting to the clinic.

### 2.2. Study Design

#### 2.2.1. Case Selection (Inclusion and Exclusion Criteria)

Medical records of all dogs and cats presented to a private small-animal practice in Berlin, Germany, from 1 January to 31 December 2021, during legally defined after-hours emergency periods, were extracted from the patient information system, categorized, and re-evaluated. In accordance with the GOT, the regular consultation hours of the veterinary practice were taken into account when determining whether a presentation occurred during an after-hours emergency period. As a result, the following after-hours periods analyzed in this study were defined as: Monday to Friday, 7 p.m. to 8 a.m.; Saturday 1 p.m. to Monday 8 a.m.; and full days on public holidays (1 January, 8 March, 2 April and 5, 1 May, 13, and 24, 3 October, and 25 December and 26, 2021).

Inclusion criteria for patients in the study were: (1) presentation of the animal in the practice or as a home visit; (2) a service provided (e.g., dispensing or sale of medication, examination, and treatment) by the veterinarian on duty; and (3) the owner was invoiced for the service. Of the original 4888 patient records, 219 were excluded because they did not involve dogs or cats (but other small animals and birds), and 90 were excluded because of missing data (date of birth or sex), resulting in a total of 4579 visit records included in the analysis. In the busy after-hours emergency setting, the level of detail in the electronic medical records varied between cases. Cases with missing essential information required for the predefined analyses (e.g., species or age) were excluded. For all included cases, each record contained at least a diagnostic entry with a final suspected diagnosis and detailed billing records documenting all performed examinations, diagnostic procedures, and administered or dispensed medications, enabling complete classification according to the predefined study variables. Additional clinical details were not consistently available in all records but were not required for the classification or analyses performed.

Each presentation of a dog or cat at a given point in time was treated as a separate case (visit), as the aim was to evaluate the overall emergency caseload rather than the longitudinal course of individual patients. Repeat presentations reflect the real clinical workload and were therefore included as separate cases. This approach mirrors common practice in human emergency medicine, where the unit of analysis is the emergency department (ED) visit rather than the individual patient, and multiple visits by the same person are counted separately [30]. The analysis was performed exclusively using a separate, anonymized study-specific dataset, which did not retain information allowing individual visits to be reliably linked to the same animal. Consequently, distinguishing first-time from repeat presentations was not possible. Each entry in the patient information system was therefore treated as a separate presentation, and the same predefined scoring system was applied to each visit.

#### 2.2.2. Data Collection

Individual records of admissions during emergency hours were extracted from a commercial patient information system (Vetera; Vetera GmbH, Eltville am Rhein, Germany), exported as portable document format (PDF) files containing the complete medical records of each patient, and subsequently reviewed by the first author. The electronic medical records were based on a standardized structured documentation template routinely used during emergency consultations. The template provided predefined sections for clinical documentation (e.g., medical history, clinical examination, organ-system-specific examination, diagnostic findings, assessment, and treatment plan), which were completed using standardized fields allowing free-text entries and predefined clinical examination categories (e.g., general condition, heart rate, respiratory rate), rather than checkboxes or selectable response options. In addition, narrative entries were used to document therapeutic measures (including medication prescriptions and discharge instructions), client communication (e.g., costs and follow-up recommendations), referral decisions, and other relevant clinical information. Billing information provided a detailed overview of all performed clinical procedures, including examinations, diagnostic tests, and administered medications.

For this study, electronic medical records were reviewed in full, and relevant clinical information was manually extracted into a structured format according to predefined variables in a Microsoft Excel for Mac (version 16.95.4, Microsoft Corporation, Redmond, WA, USA) spreadsheet. The subsequent analysis was performed exclusively using this study-specific dataset. Extracted variables included date and time of presentation, species, breed, sex, date of birth, reason for presentation, and treatment provided.

For this study, the time of entry in the patient’s medical records by medical staff was defined as the patient’s arrival at the emergency department (or the time of the home visit). The exact patient age at the date of presentation was calculated using the date of birth. Subsequently, age was categorized into species-specific age groups based on published age tables provided by Merck Sharp & Dohme (MSD, Rahway, NJ, USA) to account for species-specific differences in life expectancy and to characterize the age distribution of dogs and cats [31,32]. Variations in similar mixed breeds were grouped to form broader mixed breed classes (e.g., “European Shorthair mix”, “British Shorthair mix”).

To enable a more detailed analysis of case presentations and to assess workload patterns across shifts, the entire after-hours service period was divided into predefined time intervals for analytical purposes. The time of presentation was categorized as follows: early morning (5 a.m. to 9 a.m.), late morning (9 a.m. to 12 noon), early afternoon (12 p.m. to 3 p.m.), late afternoon (3 p.m. to 5 p.m.), evening (5 p.m. to 9 p.m.) and night (9 p.m. to 5 a.m.). Veterinarian work shifts on site were defined as day shift (9 a.m. to 4 p.m.), late shift (4 p.m. to 11 p.m.), and night shift (11 p.m. to 9 a.m.).

#### 2.2.3. Classification of Visits

Based on the full medical record information, all patients were categorized by the first author with regard to (1) the main reason for visit, (2) the type of medical care given, and (3) the triage category. In addition to the documented owner-provided reason for presentation, this retrospective classification was based on documented patients‘ medical history, physiological parameters from the clinical examination (i.e., general condition, nutritional status, mucous membranes, capillary filling time, teeth, eyes, ears, lymph nodes, head, heart, heart rate, lungs, respiratory rate, pulse, abdomen, rectal temperature and “other”) and from additional diagnostics (e.g., blood test, imaging, fecal examination). Using (1) the anamnesis, (2) the basic symptoms and disease descriptions by the veterinarian on duty, and (3) the entered suspected diagnoses, the presented cases were retrospectively categorized into (1) reason for presentation and (2) type of veterinary care performed. The classification considered previously established criteria [1,2,33]. Other studies have defined similar categories for classifying reasons for presentation to the veterinary emergency service [34,35,36,37,38].

In cases with overlapping clinical signs, each visit was assigned to a single primary category based on the overall clinical context, including history, presenting signs, and the final suspected diagnosis recorded in the medical record. Where multiple differential interpretations were possible, classification was based on the most likely underlying condition as documented by the attending veterinarian in the diagnosis field. A secondary presenting complaint was recorded in a subset of cases (*n* = 125) but was not included in the analysis presented here.

Reason for presentation was classified as no medical problem (e.g., purchase of medication), gastrointestinal tract, respiratory tract, poisoning, ear, nose and throat diseases including eyes, trauma and shock including bleeding disorders, kidney and urinary tract diseases, seizures, musculoskeletal system including lameness, cardiovascular diseases, metabolic diseases and allergies, euthanasia, and others (Table 1).

Veterinary care was classified as delivery and sale, refusal of veterinary advice, symptomatic therapy including medication, diagnostics, outpatient infusion therapy, inpatient admission, referral to a veterinary hospital, determination of death including euthanasia and resuscitation, wound care including dressing and surgery including anesthesia, and home visit (Table 2).

For both categories, namely reason for presentation and medical care (only the subcategory diagnostics), the three to four most common symptoms of each subcategory were further classified using data from the medical records (e.g., “vomiting” in gastrointestinal). This was done by evaluating the frequency of occurrence of specific, defined descriptors (e.g., “diarrhea”) in the data table. Importantly, different words describing the same issue (e.g., “dolence” or “pain”) were included in the same category.

Various triage systems exist to provide human emergency medical staff with clear guidelines for assessing clinical needs and prioritizing treatment [1,39,40]. The Manchester Triage System (MTS) is most widely used in Europe because it is considered to fit the European healthcare system better than other five-point systems [1]. The system uses five color-coded triage categories, each linked to a clinical description and a target waiting time. Two veterinary-specific triage approaches, the Veterinary Triage List (VTL) [1,2] and the Veterinary Triage System (VetTriS) [3], have also been described; however, no single universally standardized triage system is currently implemented across veterinary emergency services.

Visits were classified retrospectively by the first author using an adaptation of the MTS, which was routinely applied in clinical practice throughout the study period. Retrospective triage based on medical records is an established approach in human emergency medicine research for large-scale epidemiological studies, despite known limitations regarding data completeness, dynamic clinical changes, and under- or over-triage [39,40,41,42]. As the aim of this study was to categorize cases according to clinical urgency rather than to evaluate or validate a triage system, the MTS provided a straightforward and consistent framework for retrospective classification. Veterinary-specific triage systems (VTL [1,2] and VetTriS [3]) are cited to acknowledge existing alternatives but were not applied, as they were not routinely used at the study institution during the study period; importantly, VetTriS had not yet been published when the 2021 patient records were retrospectively analyzed and classified.

**Figure 1:**
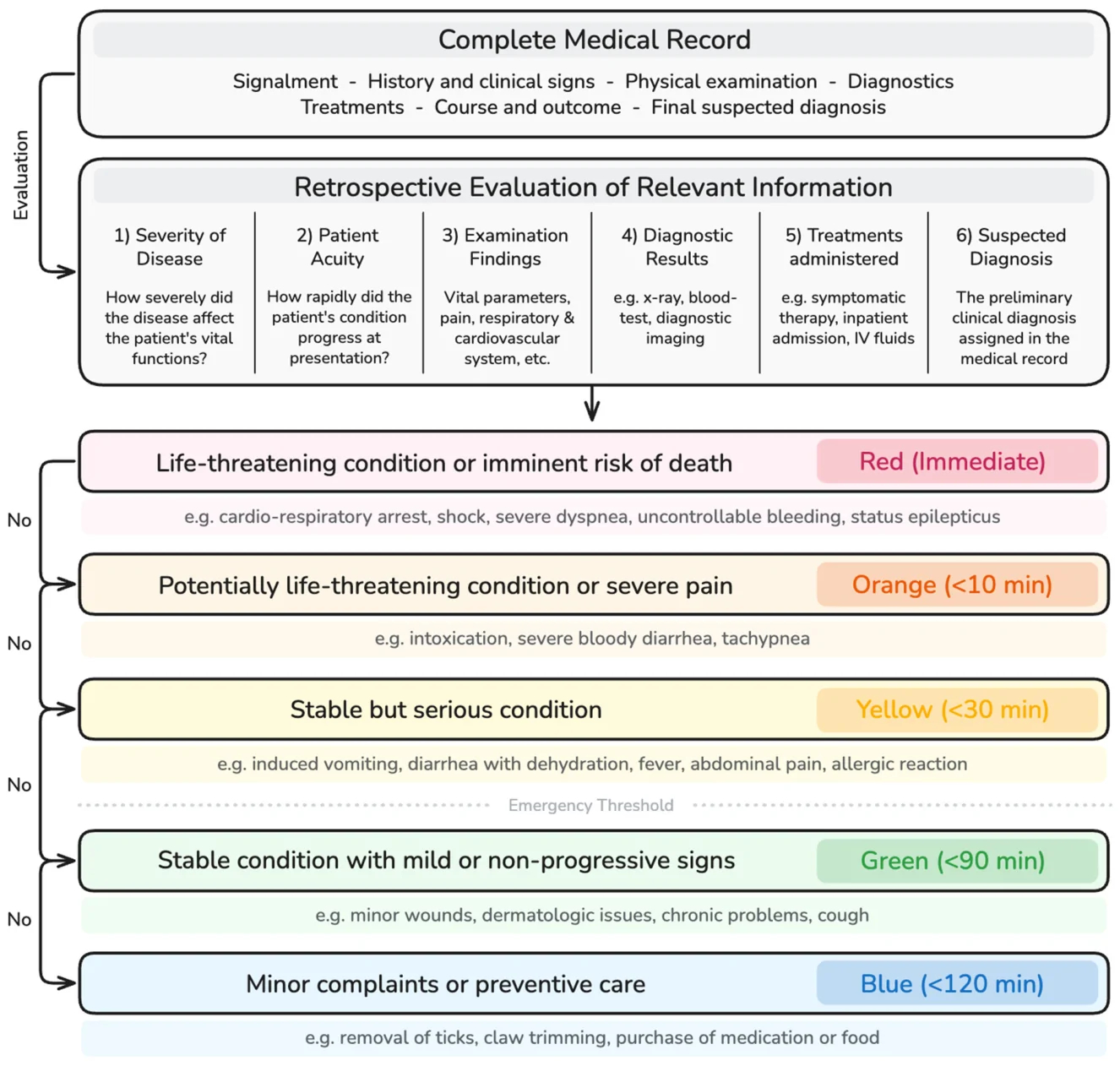
Flow chart illustrating the retrospective assignment of after-hours emergency presentations to a German private small-animal practice (*n* = 4579; 2021) to five triage categories based on medical records, including the corresponding treatment windows. Definitions of the treatment windows are provided in Table 3.

Visits were assigned a priority color code of red if considered life-threatening or at imminent risk of death, orange if considered potentially life-threatening or associated with severe pain, yellow if considered stable but serious, green if considered stable with mild or non-progressive clinical signs, and blue if presenting with minor complaints or for preventive care. Classification was based on (1) the severity of the disease described in the medical record, (2) the acuity of the patient, (3) findings of the general examination, (4) diagnostic results, (5) administered treatments, and (6) the final suspected diagnosis to retrospectively categorize the medical urgency of the visit (Figure 1). Each visit was considered as a whole, and all relevant information documented for that visit was taken into account when assigning the triage category. Thus, the classification was not based on presenting complaint alone but incorporated the overall clinical information available in the medical record, including patient characteristics, clinical findings, diagnostics, treatment, and final suspected diagnosis.

All visits assigned to the red, orange, and yellow triage categories were classified as true emergencies, whereas those assigned to the green and blue categories were classified as non-emergencies (Table 3).

### 2.3. Statistical Methods

Statistical analyses were performed using Python 3.8 (Python Software Foundation, Wilmington, DE, USA) for data handling, cleaning, and preliminary visualization. During data preprocessing, spelling inconsistencies were corrected, and variables were appropriately recoded (e.g., categorical variables transformed into numerical codes). Descriptive statistics, including frequencies, were calculated for all categorical variables (animal species, breed, sex, and age group) using IBM SPSS Statistics software (version 29.0.2.0; IBM Corporation^©^, Armonk, NY, USA). Measures of central tendency (mean and median) and variability (standard deviation, minimum, and maximum) were calculated for the quantitative (numerical) variable age. Each emergency presentation was considered a separate case to reflect the actual clinical workload of the emergency service. As individual patients could contribute more than one emergency presentation during the study period, observations were not fully independent. Therefore, this study was designed as a descriptive analysis of the emergency caseload in dogs and cats. Accordingly, no inferential statistical analyses or hypothesis testing were performed, and no statistical comparisons between species were made. Final graphical visualizations were created using Tableau 2024.01 (Salesforce Inc., Seattle, WA, USA) and Excalidraw (Excalidraw s.r.o., Brno, South Moravia, Czech Republic; https://excalidraw.com, accessed on 30 August 2026).

## 3. Results

### 3.1. Patient Profile

**Figure 2:**
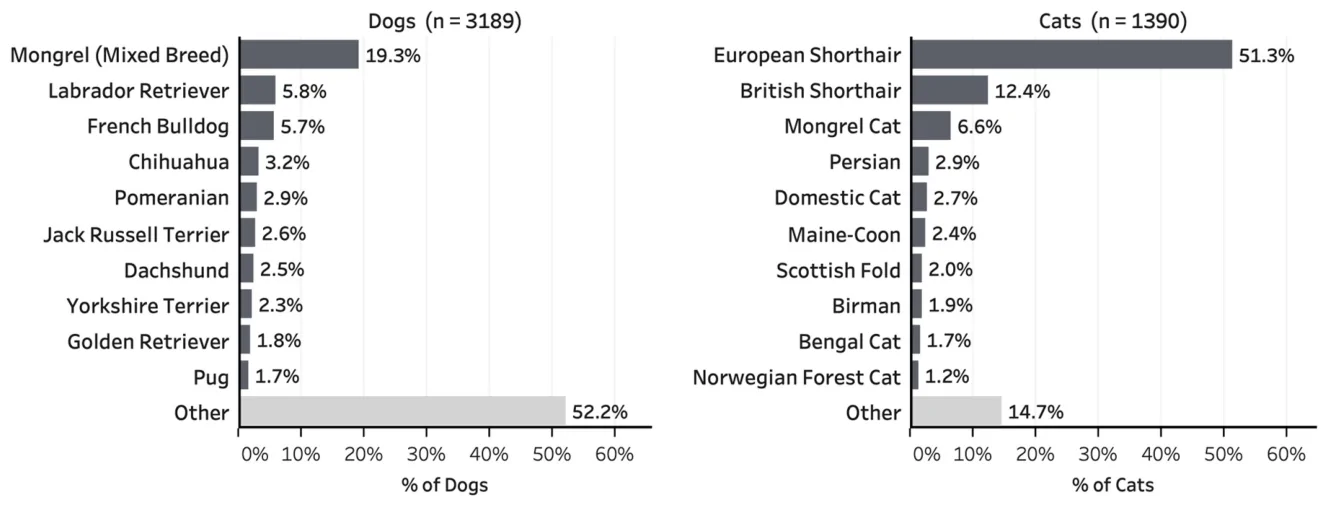
Distribution of the most common dog and cat breeds presented after hours to a German private small-animal practice (*n* = 4579; 2021).

The search of the digital database resulted in a total of 4579 cases presented during after-hours periods in 2021, of which 3189 (69.6%) were dogs, and 1390 (30.4%) were cats. The most frequent dog breeds were mongrel (mixed breed) dogs (19.3%), Labrador Retrievers (5.8%), and French Bulldogs (5.7%). Among cats, European Shorthair (51.3%) and British Shorthair (12.4%) were the most common (Figure 2). Of all patients, 2492 (54.4%) were males and 2087 (45.6%) were females.

Age ranged from 4 weeks to 25.2 years in dogs (median: 3.50) and 8 weeks to 25.8 years in cats (median: 7.58). Many patients were less than one year old (1068; 23.3%) at the time of emergency presentation. Cats had a higher median age, and the proportion of older cats (age 7+) was higher (52.3%) than in dogs (32.9%). Although the proportion of the juvenile age group was similar across both species, young dogs (67.1%) had a higher proportion than young cats (47.6%) (Table 4).

### 3.2. Days and Times

The most frequent patient presentations to the after-hours emergency service occurred in spring (28.0%) and summer (26.3%). The months May (10.9%) and April (9.2%) were the busiest. The weekdays with the highest attendance were Sunday (30.1%) and Saturday (24.0%). The most common proportion of presentations occurred during the night (9 p.m. to 5 a.m.) with 43.2%, and in the evening (5 p.m. to 9 p.m.) with 25.6%. The most frequented time of day was late shift (4 p.m. to 11 p.m.) with 47.3%, followed by the night shift (11 p.m. to 9 a.m.) with 29.9%.

**Figure 3:**
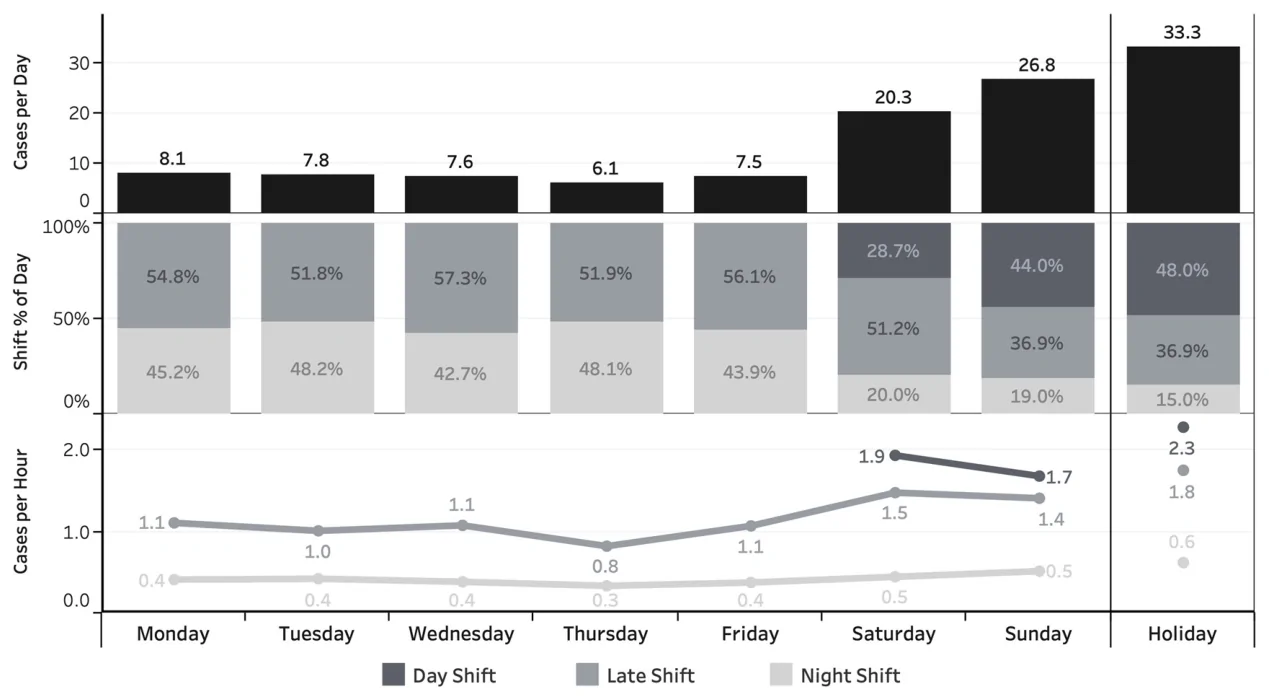
Average daily caseload (top), distribution across work shifts (middle), and hourly distribution of presentations (bottom) in dogs and cats presented after hours to a German private small-animal practice (*n* = 4579; 2021).

Holidays and Sundays had the highest average number of cases per day (2.3 cases per hour on holidays) during the day shift. On Saturday, 51.2% of presentations occurred during the late shift, yet the day shift was visited more frequently, with 1.9 visits per hour. During the week (Monday to Friday), cases per hour were relatively evenly distributed across the late and night shifts (Figure 3).

We observed a connection between seasonal occurrence and diseases, e.g., poisoning cases were higher in April and December, and ear, nose, and throat diseases were more prevalent in June than in other months.

### 3.3. Reasons for Presentation

The most common reasons for presentation among all patients who visited the emergency service were gastrointestinal tract diseases (31.2%), poisoning (11.4%), and musculoskeletal diseases, including lameness (11.4%). Animals mostly presented with vomiting, diarrhea, and inappetence as underlying symptoms. The most frequently used chief complaint descriptors for each category are listed in Table 5.

**Figure 4:**
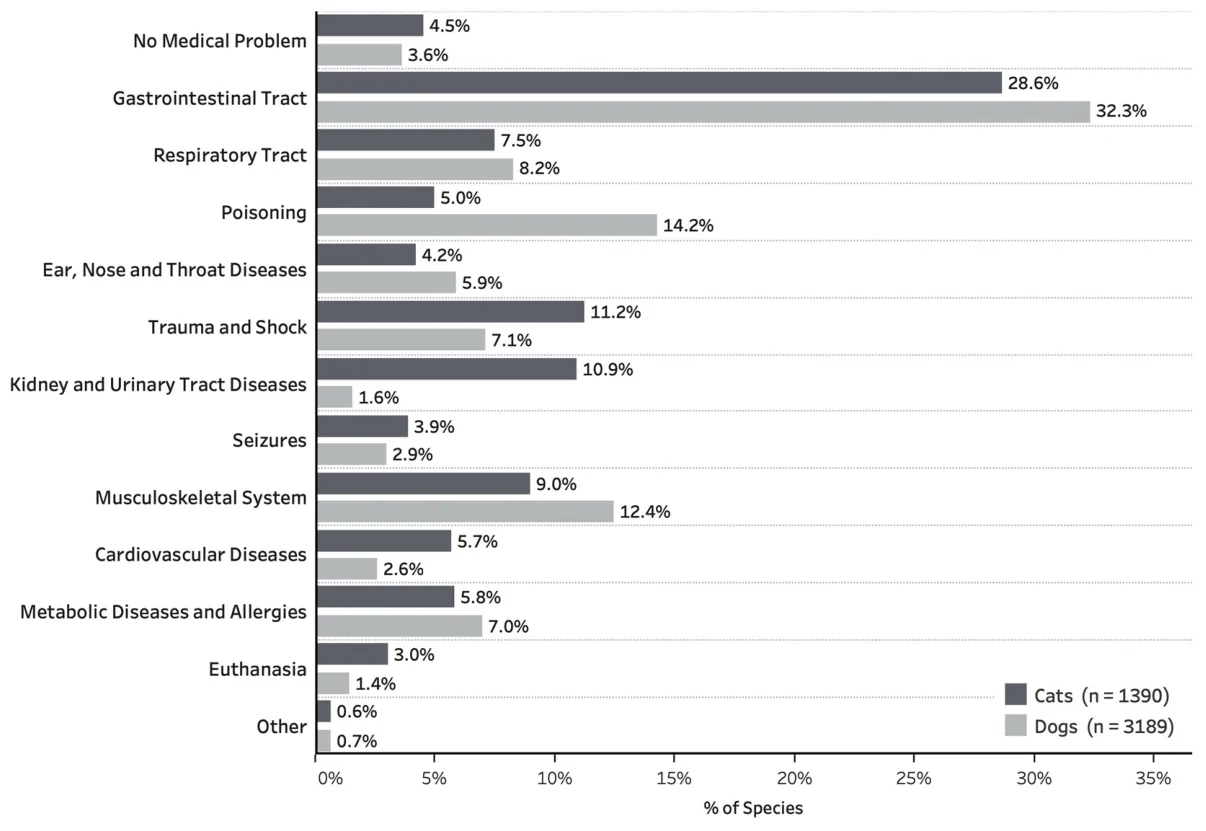
Species-specific distribution of reasons for presentation in dogs and cats presented after hours to a German private small-animal practice (*n* = 4579; 2021).

Causes of emergency visits in dogs were mainly due to gastrointestinal disorders (32.3%), poisoning (14.2%), and musculoskeletal disorders (12.4%). In cats, mostly gastrointestinal disorders (28.6%), trauma and shock (11.2%), and kidney and urinary tract diseases (10.9%) were identified (Figure 4).

### 3.4. Veterinary Care

**Figure 5:**
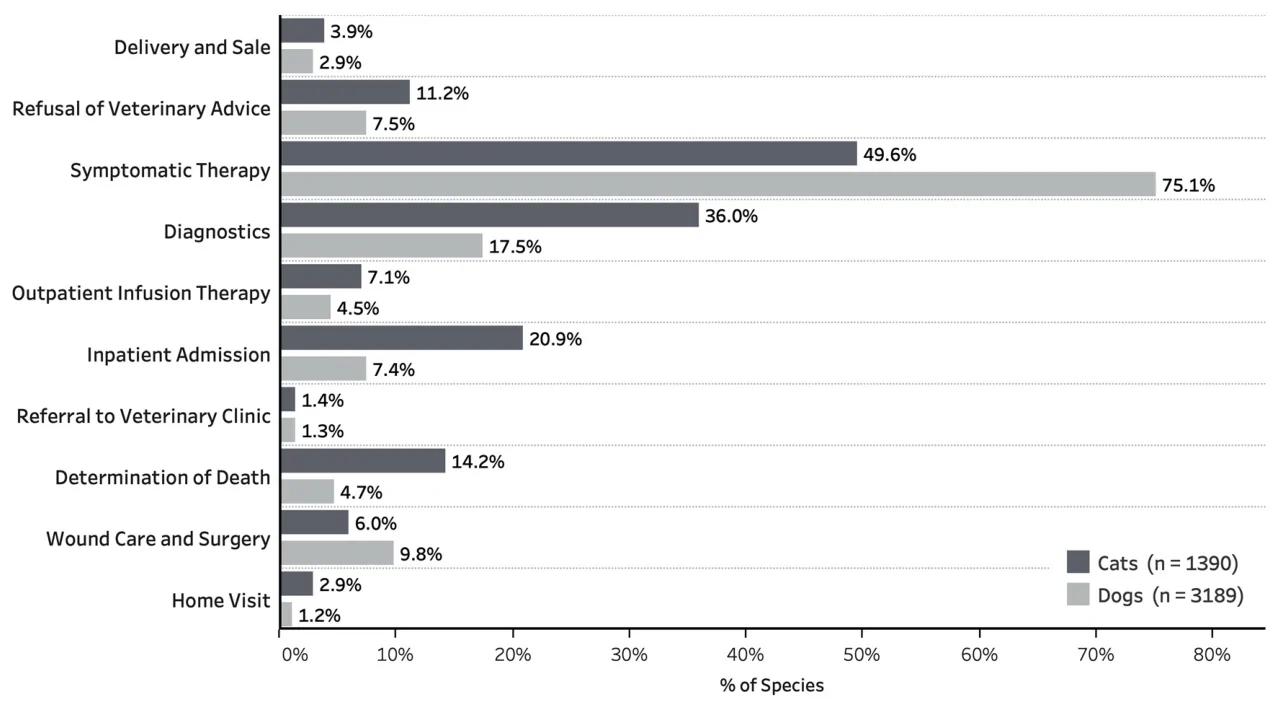
Species-specific distribution of medical management categories in dogs and cats presented after hours to a German private small-animal practice (*n* = 4579; 2021).

Across both species, the most common types of veterinary care were symptomatic treatment (67.4%), diagnostics (23.1%), and hospitalization (11.5%). The proportion of patients who received symptomatic treatment was higher in dogs (75.1%) than in cats (49.6%). Wound care or surgery was provided to 9.8% of dogs compared with 6.0% of cats, whereas outpatient infusion therapy (7.1%) and home visits (2.9%) were more common among cats. Also, further diagnostic procedures (36.0%) and hospitalization (20.9%) were more frequently recorded among cats than dogs, while death was recorded ~three times as frequently among cats by the end of the visit (14.2%). Interestingly, cat owners often refused medical advice (11.2%). Notably, only 1.3% of dogs and 1.4% of cats were referred to a veterinary hospital (Figure 5).

The presentation categories, such as poisoning, gastrointestinal tract, and ear, nose, and throat diseases including eyes, were more frequently treated symptomatically than symptoms in other categories. Animals suffering from poisoning, seizures, trauma, and shock, as well as kidney diseases, were more often hospitalized than those with other medical conditions. Infusion therapy was used more commonly in cases with gastrointestinal tract disease and kidney and urinary tract disease than in cases with other symptoms. Musculoskeletal disorders received wound care and surgery more than other treatments. Animals with cardiovascular disease were more often observed to be euthanized or treated during a home visit. Euthanasia was performed predominantly during a home visit rather than during other services.

Diagnostic procedures such as X-ray, blood test, and ultrasound were more common in patients who presented for seizures, gastrointestinal disorders, trauma and shock, renal disease, and cardiovascular disease than for other symptoms. In the diagnostic category, the most common descriptors for medical care in the compiled data table after reviewing all medical records of the practice data were (1) X-ray, (2) blood test, (3) ultrasound, and (4) blood pressure (Table 5).

### 3.5. Triage

The most common triage level was green (standard) (2192; 47.9%), followed by yellow (urgent) (1048; 22.9%) and red (immediate) (581; 12.7%). Taking blue cases (non-urgent) (314; 6.9%) and green cases into account together, over 50% of all cases were classified as non-emergencies. Of all cases, 22.4% required immediate or very urgent treatment (red and orange cases combined). With 22.9% of yellow cases (urgent) being presented, true emergencies accounted for 45.3% of all patient presentations.

**Figure 6:**
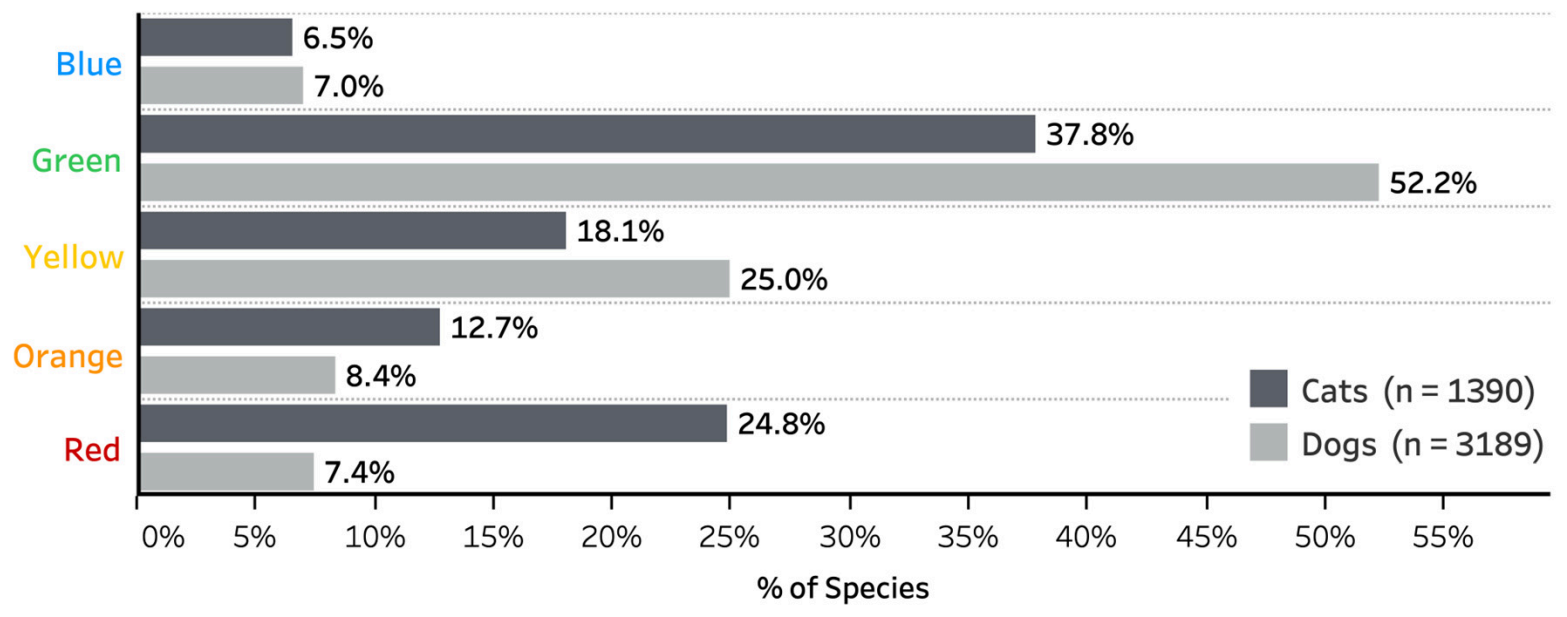
Species-specific distribution of triage categories in dogs and cats presented after hours to a German private small-animal practice (*n* = 4579; 2021).

Cats were more often assigned to true emergencies (55.6%), taking yellow (urgent), orange (very urgent) and red (immediate) triage levels into account together. Non-emergency cases were classified for 44.3% of cat presentations. Dogs were mostly assigned to green (52.2%) and yellow (25.0%) triage levels. Cat presentations (red triage, 24.8%) were classified as life-threatening emergencies ~three times as often as dog presentations (red triage, 7.4%) (Figure 6). Red triage presented mainly as cardiovascular disease and euthanasia in dogs (18.4%) and as trauma and shock in cats (23.1%). More severe triage categories were more frequently observed among older animals in both species.

**Figure 7:**
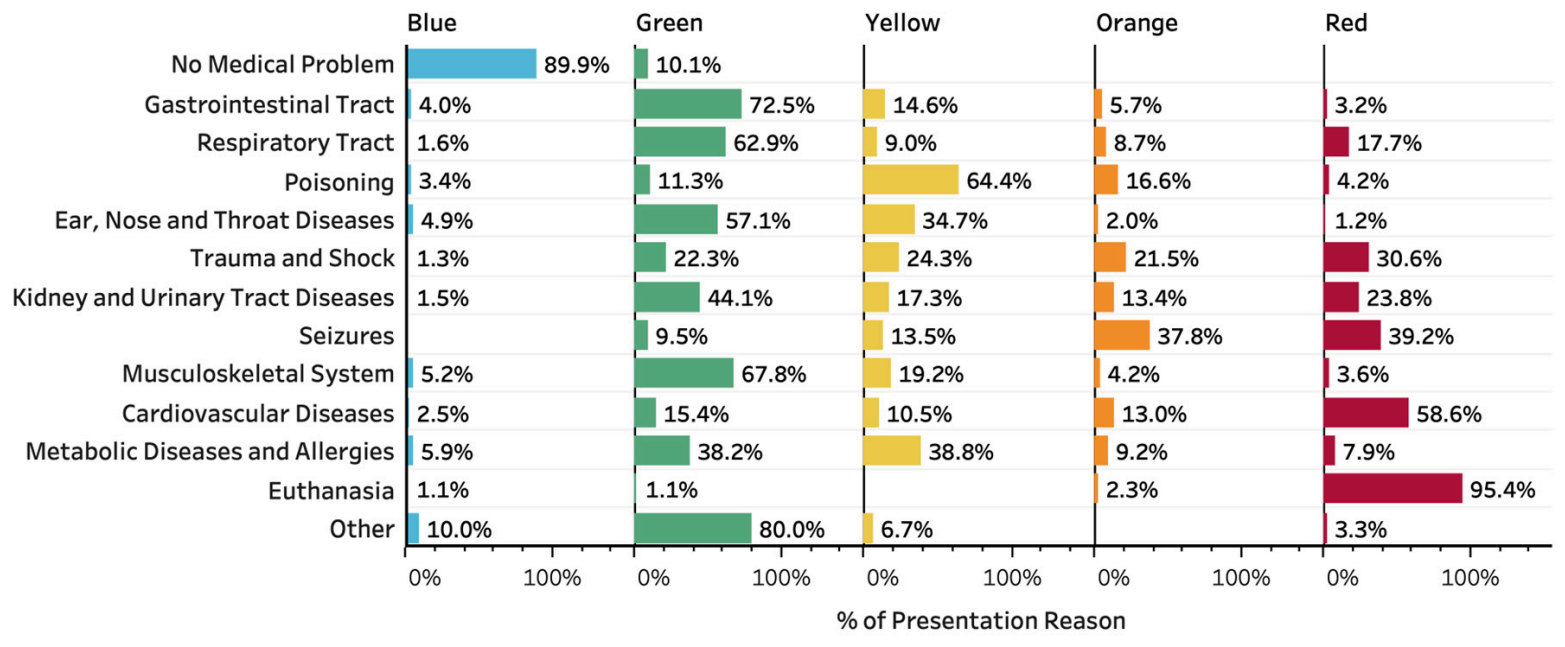
Distribution of triage categories according to reasons for presentation in dogs and cats presented after hours to a German private small-animal practice (*n* = 4579; 2021). Euthanasia cases were classified by clinical urgency at presentation: immediate euthanasia due to severe suffering was assigned to red, indicated but non-immediate cases to orange, and non-euthanized cases were classified according to clinical status (blue/green).

In triage distribution, gastrointestinal tract presentations were mainly classified as green (72.5%), while poisonings were often classified as yellow (64.4%) and orange (16.6%). Respiratory problems were often classified as green (62.9%) and red (17.7%) depending on the type of illness (e.g., mild coughing vs. dyspnea). Also, categories like trauma and shock, kidney and urinary tract disease, seizures, heart disease, and euthanasia accounted for a larger proportion of triage red compared to the other triage levels (Figure 7).

Visits classified as red and orange frequently involved diagnostic procedures, in-house hospitalization, or referral to a veterinary clinic. Euthanasia or determination of death and home visits were predominantly recorded among visits classified as red. Symptomatic therapy was mostly classified as green and yellow. Refusal of veterinary advice and outpatient infusion therapy were more often classified as orange cases than at the other triage levels. Cases requiring wound management, including wound care procedures, surgery, and anesthesia, were predominantly assigned to the yellow and orange triage categories.

**Figure 8:**
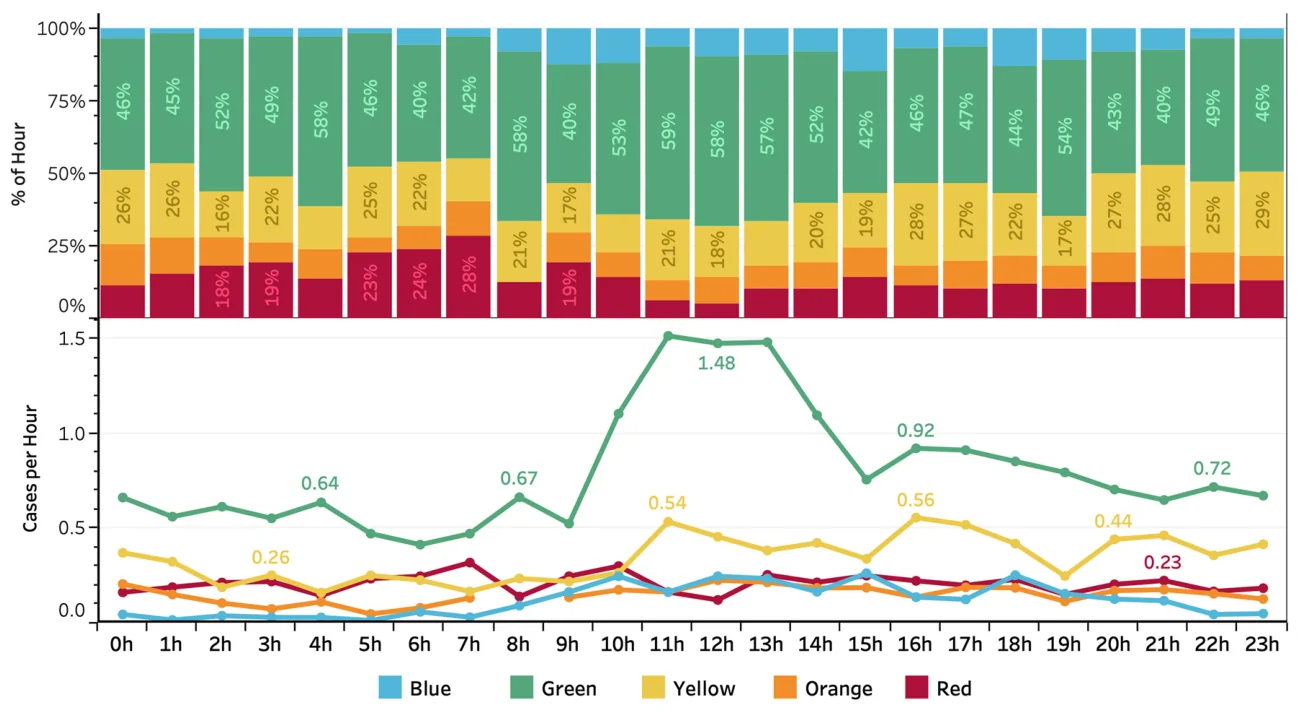
Hourly distribution of triage levels during after-hours periods (nights, weekends, and public holidays) (**top**) and corresponding average caseload per hour (**bottom**) in dogs and cats presented to a German private small-animal practice (*n* = 4579; 2021).

Visits with blue triage were mainly presented from 10 a.m. onward, with a stable level until 6 p.m. After that time, fewer patients presented, with rare blue cases presenting at night. Triage green maintained a steady level of 40–60% of presentations per hour and is considered the most dominant triage during the very high frequency between 10 a.m. and 2 p.m. The busiest time of day was driven by the green triage, with a marked increase in showings per hour between 9 and 11 a.m., resulting in a doubling of presentation volume. From 11 a.m. onward, the yellow triage was more frequent, with a volume of 13–29% per hour. With a constant level as the second-most-frequent triage, it was more common later in the evening. Visits with the triage red had their highest peak at 7 a.m.; the least likely time for this triage was 12 noon. Orange visits were presented most prominently early in the morning (Figure 8).

## 4. Discussion

### 4.1. Study Objectives and Literature Context

Against the backdrop of increasing demands on after-hours veterinary emergency services in Germany and the growing concern that non-emergency presentations contribute to service overload hours [16,17,18] as described in human emergency medicine [4,5,18,19,20,21,22,23,24,25,26,27,28,29], this study aimed to characterize the after-hours emergency caseload of a 24/7 small-animal practice in Berlin in 2021 and to determine the proportion of true emergencies and non-emergencies using a retrospective triage classification. This study addresses a gap in the limited literature on small-animal emergency caseloads in Germany and provides novel data on temporal patterns, patient characteristics, reasons for presentation, medical management, and emergency service utilization. Only a few studies from Germany either analyzed the general small-animal practice population [43] or focused on selected emergency conditions such as poisoning [44,45]. Internationally, several publications described the overall emergency caseload for small animals [34,35,37,38,46,47,48], while others focus on case-specific analyses of conditions presented during emergency service [44,45,49,50,51,52,53,54,55,56,57,58,59,60,61,62,63]. Some studies have examined temporal patterns in emergency service utilization, including time of day and external temporal factors such as holidays, lunar phases, and significant social events [36,64,65,66,67]. Our findings may inform future research, support emergency service planning, and contribute to a better understanding of the distribution of true emergencies and non-emergency presentations in after-hours veterinary care both in Germany and other countries.

### 4.2. Characteristics of After-Hours Emergency Visits

In our study, nearly twice as many dogs as cats were presented, despite cats outnumbering dogs in German households in the year 2021 (16.7 million cats vs. 10.3 million dogs) [68]. Other international studies also reported substantially more dogs than cats [34,48,65,66]. The most frequently presented dog and cat breeds reflect both overall breed popularity [69,70] and the general small-animal practice population in Germany [43]. Specific case studies on poisonings in dogs and cats in emergency settings in Germany reported similar breeds [44,45].

The higher caseload per day observed during weekends and particularly on public holidays is explained by the longer and additional periods covered by after-hours emergency services, including the availability of daytime emergency shifts that are not offered on weekdays. Late shifts showed increased caseloads per hour on weekends and public holidays, compared with weekdays, while day shifts represented the busiest periods overall, especially on public holidays. In contrast, night shifts showed only minor differences between weekdays, weekends, and public holidays in terms of caseload per hour, suggesting a relatively stable and predictable workload during these hours. Therefore, night shifts appear to contribute less to the increased caseloads observed on weekends and public holidays, whereas the additional daytime and late-shift visits during these periods may represent the main drivers of increased staffing demands. The increased caseloads in April and May may be related to the higher number of public holidays. During summer, longer daylight hours, school holidays, and increased outdoor activities may contribute to more presentations, particularly during late shifts. These temporal patterns are consistent with previous studies [36,66] and may be influenced by greater owner availability outside regular working hours and reduced access to routine veterinary care during these periods. These findings may be provided for future workforce planning, as staffing levels and emergency duty scheduling could potentially be adapted to periods of increased workload.

Gastrointestinal disease was the most common reason for emergency visits in both species; this finding is consistent with previous studies in small-animal emergency settings [35,37,38,48]. Presenting complaints such as diarrhea and vomiting are readily apparent to owners and often prompt veterinary consultations [71]. In dogs, the other leading causes of emergency presentations were poisoning and musculoskeletal disorders, including lameness. Poisonings are a common reason for visits to small-animal emergency clinics, with dogs being the species most frequently affected by accidental ingestion of toxins [45]. Lameness is perceived by owners as distressing and a decreased quality of life for the animal; it is therefore a common presentation that emergency clinicians must manage alongside true emergencies [63]. In feline presentations, trauma and shock were the second most common reason in our study. Trauma is a leading cause of morbidity and mortality in cats and represents a highly prevalent reason for emergency presentation in both cats and dogs [34,72]. The urban environment may contribute to the occurrence of trauma cases, as previous studies have reported a higher prevalence of high-rise syndrome (HRS) in urban areas [73,74]. In our study, HRS was the second most common descriptor within the trauma and shock category; however, its specific contribution to the observed cases was not further investigated and therefore remains speculative. Kidney and urinary tract diseases were the third most common reason for feline presentation. Lower urinary tract signs (LUTS), particularly feline urethral obstruction (FUO), are potentially life-threatening and require immediate treatment [75,76]. Previous studies also identify urinary disorders as a leading cause of emergency care in cats [34,38,75]. From a clinical and organizational perspective, the observed disease spectrum reflects the broad range of conditions encountered in after-hours emergency practice, while also demonstrating that certain clinical presentations are considerably more prevalent. Emergency clinicians should therefore be prepared to manage a wide variety of common conditions, especially gastrointestinal, toxicological, musculoskeletal, traumatic, and urinary tract diseases. Respectively, owner education should focus on recognizing signs associated with common presentations, particularly gastrointestinal disorders, that require immediate emergency care versus those that can safely wait for a regular veterinary consultation.

Across both species, symptomatic treatment was the most common intervention in our study population, reflecting the high proportion of non-urgent presentations, especially the above-highlighted gastrointestinal diseases. However, cats required diagnostic procedures, hospitalization, and end-of-life care more often than dogs, consistent with their higher proportion of potentially life-threatening presentations. The frequent use of diagnostics, particularly radiography, blood testing, and ultrasonography, as well as the management of cases requiring fluid therapy or inpatient care, highlights the importance of maintaining essential diagnostic and treatment capacities in after-hours services. Therefore, emergency practices should ensure the availability of basic diagnostic equipment, monitoring capabilities, and personnel trained in stabilization and initial management of critical patients. The low referral rate suggests that many cases can be managed within the practice; nevertheless, predefined referral pathways remain essential for patients requiring specialized expertise or advanced diagnostics. Additionally, owner decisions, including refusal of recommended diagnostics or treatment, emphasize the importance of transparent communication regarding disease severity, treatment options, and expected costs during emergency consultations.

### 4.3. Medical Urgency of After-Hours Visits

In human emergency departments, visits have steadily increased, particularly among patients who do not require urgent care [22,25,26,28]. According to estimates by the German Federal Chamber of Veterinarians, ~80% of services provided during after-hours emergency are not true emergencies and therefore generate only limited revenue [77]. After-hours presentation of non-urgent cases presents an ethical challenge, as limited staffing and resources, increased costs, and the potential diversion of attention from true emergencies must be weighed against the benefits of deferring care to regular working hours [17]. This is reflected in our study, in which more than half of all after-hours presentations were classified as non-emergencies, with 47.9% assigned to the green and 6.9% to the blue triage category. From a clinical perspective, these findings suggest that a substantial proportion of patients presenting to the after-hours service may have been suitable for management during regular consultation hours in primary care practices. This was particularly evident for non-urgent cases classified as blue, for which emergency presentation was likely not required.

The temporal distribution of triage categories showed that non-urgent presentations (blue and green) predominated during daytime hours, particularly between late morning and early afternoon, when the overall workload was highest. In contrast, higher urgency categories showed distinct patterns, with orange and red cases occurring more frequently in the early morning, with red cases peaking between 5 and 7 a.m. Still, even during the periods with the highest proportion of urgent cases, non-emergency presentations accounted for at least 40% of hourly presentations, highlighting the persistent challenge of managing lower-acuity cases within after-hours emergency services. Additionally, these findings suggest that between midnight and early morning when emergency veterinarians often work without additional support staff, adequate staffing and preparedness are essential to manage potentially critical cases. At the same time, the high number of non-urgent presentations during daytime hours may further contribute to workload and resource demands in after-hours services.

Non-emergency presentations were predominantly driven by dogs, with nearly 60% of canine cases classified as non-emergencies. This finding may be explained by the observation that the two most common reasons for canine emergency presentations, gastrointestinal and musculoskeletal disorders, including lameness, were also predominantly classified as non-urgent. More than 75% of gastrointestinal presentations and over 70% of musculoskeletal cases were classified as green or blue. True emergencies were more common in cats, which again may be explained by the more life-threatening nature of two of their most common reasons for presentation. More than 75% of trauma and shock, and 50% of kidney and urinary tract diseases were classified as true emergencies (yellow, orange, or red). The higher proportion of severe triage categories in cats compared with dogs highlights species-specific differences in emergency presentation patterns and emphasizes the need for careful initial assessment. Among euthanasia cases, 95.4% were classified as red, partly because immediate euthanasia for severe suffering was itself a red-category criterion; however, only 3.0% of cats and 1.4% of dogs received euthanasia.

These findings underline the importance of structured triage use in clinical practice, appropriate diagnostic and treatment capabilities, and adapted staffing during peak periods with presentations of urgent cases. Improved telephone triage and owner education may further help guide non-urgent cases toward appropriate care pathways while ensuring timely management of true emergencies.

### 4.4. Potential Factors Influencing After-Hours Emergency Visits

Several factors described in the literature may potentially influence after-hours emergency presentations. Although these factors were not examined in the present study and their contribution therefore remains speculative, discussing them provides context for interpreting the observed patterns and highlights potential determinants to be addressed in future studies.

Environmental and population-related factors may contribute to specific presentation patterns. Urban environments may increase exposure to hazards such as trauma [73] or toxin sources [78,79], while breed-related predispositions may influence the occurrence of certain disorders [80,81,82,83,84,85,86]. The high proportion of young animals observed in our cohort may reflect increased acquisition of puppies and kittens during the COVID-19 pandemic [87,88,89], as well as their higher susceptibility to infectious and gastrointestinal diseases [90]. Species-specific owner behavior may also affect presentation patterns [71,91,92], as cats may be presented later due to their tendency to mask clinical signs [93], potentially contributing to more severe disease at presentation [48,94]. Owners frequently misjudge urgency [63,92], and owner-reported complaints in emergency settings often poorly correlate with final diagnoses [35].

Seasonal factors may further influence emergency presentations, for example, through increased chocolate exposure during Easter and Christmas periods or summer-related conditions such as grass-awn-associated diseases.

COVID-19-related changes in pet ownership and owner behavior [71,89,91,92,95] may have further influenced healthcare-seeking patterns during the study period. For example, increased prevalence of urethral obstructions during the COVID-19 pandemic may reflect environmental stressors [61]. Also, COVID-19-pandemic-related anxiety [96] may have increased overcautious presentations.

Finally, socioeconomic factors, including financial concerns [97] and limited access to veterinary care, may affect decisions regarding emergency consultation, diagnostics, and hospitalization, particularly in the after-hours setting where costs are typically higher. Delayed presentation may reflect owners seeking veterinary care later than human medical care due to financial or emotional factors, or an underestimation of disease severity [1]. Pet insurance coverage may represent an additional socioeconomic factor influencing owners’ decisions regarding veterinary care, particularly in emergency settings where costs can be substantial. The available literature suggests that insurance may reduce financial barriers to veterinary care, increase owners’ willingness to pursue advanced diagnostics or treatment [98], and decrease the likelihood of economic euthanasia in emergency settings [99]. However, the extent to which insurance status affects after-hours veterinary presentations or clinical urgency remains unclear. Future studies incorporating information on insurance status may help to clarify whether and to what extent pet insurance influences emergency service utilization, owners’ treatment decisions, and the pursuit of additional diagnostics, referral, or treatment.

### 4.5. Limitations

Several limitations should be considered when interpreting the study findings. First, case classification and retrospective triage were performed by a single reviewer without independent verification or inter-rater reliability assessment. Although standardized definitions and protocols based on previously established criteria were applied consistently to all cases to minimize subjectivity, the absence of independent review or consensus-based classification may have introduced subjectivity and classification bias. Therefore, the consistency and reproducibility of the retrospective classification could not be independently assessed, and the classification should be interpreted as a descriptive categorization of the included visits rather than as a validated assessment of triage performance.

Furthermore, although the medical record template was standardized, the completeness and level of detail of the free-text entries varied among the ~20 attending veterinarians. Owner-reported information, such as age or breed, may be inaccurate, particularly for animals from welfare organizations. Retrospective triage based on medical records may additionally limit the accuracy of urgency categorization, as real-time clinical dynamics, subtle clinical signs, and disease progression cannot be fully captured.

A further limitation is that the MTS was originally developed for human medicine and has not been formally validated for veterinary patients; therefore, it may not fully account for species-specific differences in clinical presentation and disease progression [1]. The MTS was selected because it provided a practical and familiar framework for structured retrospective classification. The aim of this study was not to validate a triage system but to categorize cases according to medical urgency. Future studies could reanalyze or validate these findings using veterinary-specific systems, such as the VTL [1,2] or the VetTriS [3]. For example, including a “pink” category for euthanasia cases could further improve classification.

A further limitation concerns the potential circularity inherent in the retrospective classification. Because diagnostic findings, treatments, and the final suspected diagnosis contributed to the classification, relationships between triage categories and these variables should be interpreted as characteristics of the retrospectively classified visits rather than as independent associations. This potential circularity is greatest in extreme cases, such as immediate euthanasia of animals with severe suffering, which was included among the criteria for assignment to the red triage category.

Repeat visits by the same patient were analyzed as separate cases because the study focused on emergency presentations and the overall clinical workload rather than individual animal progressions over time. As the analysis was based on a separate anonymized dataset, individual visits could not be reliably linked to the same animal, and clustering of repeated visits could therefore not be assessed. However, because the analyses were purely descriptive, no inferential statistical associations or causal relationships were evaluated. This potential clustering should therefore be considered when interpreting the reported visit-level frequencies.

Finally, data were collected in 2021 during the COVID-19 pandemic, which may have affected owner behavior, access to veterinary care, and emergency caseloads. Consequently, presenting complaints, medical care, and triage patterns observed may not fully reflect non-pandemic conditions, and caution is warranted when generalizing these findings.

Despite the large dataset and detailed characterization of cases within a high-volume urban 24/7 small-animal emergency practice in Germany, findings should be interpreted within this specific clinical and geographical context. The generalizability to other practice types, regions, or healthcare systems should be done with caution since local contextual factors, including client demographics, practice structure, and available diagnostic and inpatient resources, may have influenced the observed case distribution.

## 5. Conclusions

Epidemiological data on veterinary emergency caseloads in the small-animal sector in Germany remain limited, despite increasing demands for after-hours veterinary care. The majority of visits were classified as non-emergency and managed symptomatically; however, cats presented more often with life-threatening conditions than dogs. Dogs were presented more often than cats, with non-emergent gastrointestinal disease being the most common reason for presentation in both species. After-hours caseloads were highest during spring and summer, particularly in April and May, as well as on holiday and weekend day shifts, indicating predictable periods of increased service demand. Diagnostic imaging, laboratory testing, and hospitalization were common, indicating the need for comprehensive emergency service capabilities. Together, these findings provide important insights into the current after-hours emergency caseload in a German small-animal practice and highlight the substantial burden of non-emergencies on after-hours veterinary emergency services. Improving owner education regarding appropriate emergency service utilization, optimizing resource allocation within emergency practices, and implementing structured on-site triage are essential to ensure timely care for critically ill patients while reducing unnecessary pressure on emergency services. However, as this was a single-center retrospective study, the findings have limited generalizability. Further multicenter prospective research is needed to provide more robust evidence on emergency presentations and their clinical urgency in small-animal practice.

## Figures and Tables

**Table 1:** Classification of presenting complaints in dogs and cats presented after hours to a private small-animal practice in Germany (*n* = 4579; 2021).

Presentation Category	Description	Presenting Complaints and Diseases (Examples)
**No medical problem**	No noticeable health issues or sale of medication/food (with/without the animal present)	Purchase of medication, food, cat milk, neck collars, pill dispensers, tick tweezers, etc.; removal of a tick, trim claws
**Gastrointestinal tract**	Digestive system disorders	Vomiting, diarrhea, fecal impaction, abdominal pain, nausea, dental disease, weight loss, gastroenteritis, stomach overload, loss of appetite
**Respiratory tract**	Respiratory system disorders	Shortness of breath, cough, laryngitis, rapid breathing, cat flu, reverse sneezing, bronchitis, pneumonia
**Poisoning ^1^**	Poisoning or toxic exposure	Induced vomiting (ingestion of foreign objects of any kind, e.g., socks, linen, mice, poison bait), intoxication (chocolate, onions, grapes, xylitol, coumarin, human drugs, medications such as ibuprofen), hypersalivation, apathy, hyperesthesia, somnolence, muscle tremors
**Ear, nose and throat diseases including eyes**	Ear, nose, throat, and eye disorders	Otitis, conjunctivitis, corneal injuries, eye inflammation, itchy eyes and ears, glaucoma, grass seeds in the ear, foreign bodies in the nose, nasal discharge
**Trauma and shock including bleeding disorders**	Injuries, shock, or bleeding disorders	Injuries caused by wild boar attacks, HBC, HRS, bite wounds, pain, wound treatment, cuts, stick injuries, trauma after falls, bleeding of any kind
**Kidney and urinary tract diseases**	Kidney and urinary tract disorders	Cystitis, strangury, bladder tumors, anuria, prostatic hyperplasia, LUTS, polyuria and polydipsia, vaginal discharge, renal insufficiency, hematuria
**Seizures ^2^**	Seizures or convulsions	Epileptiform seizures, vestibular syndrome, hypersalivation, tremor and muscle tremors, status epilepticus, syncope, ataxia, nystagmus, balance disorders, convulsions
**Musculoskeletal system including lameness**	Musculoskeletal disorders, including lameness	Lameness, musculoskeletal pain, claw avulsion, cruciate ligament rupture, ball horn avulsion, wound treatment and dressing changes, fractures, joint swelling, patellar luxation, paresis, foreign bodies between the toes, awns in the paw
**Cardiovascular diseases**	Heart and blood vessel disorders	Aortic thrombosis, heart failure, cardiac arrhythmias, agony, hypertension, syncope, pericardial effusion, heat stroke
**Metabolic diseases and allergies**	Metabolic disorders or allergies	Fever, allergic reactions, anaphylactic shock, pancreatitis, itching, diabetes, urticaria, hot spot, Cushing’s disease, insect bites, hyperthyroidism
**Euthanasia**	Euthanasia performed	Euthanasia (including home visits)
**Other**	Other or unclassified health issues	Pain of unknown origin, tremors, malaise, clouding of consciousness, weakness, penile prolapse, behavioral changes

Legend: HBC = hit by car; HRS = high-rise syndrome; LUTS = lower urinary tract signs. ^1^ Poisoning was defined as the ingestion of any foreign substances or materials not intended for consumption by the animal. ^2^ This category includes neurological disorders. Cases were classified based on the final suspected diagnosis and assigned to a single category according to the primary presenting complaint, despite overlap in clinical signs across categories.

**Table 2:** Classification of treatment and patient management in dogs and cats presented after hours to a German private small-animal practice (*n* = 4579; 2021).

Veterinary Care Category	Description	Performed Veterinary Care (Examples)
**Delivery and sale**	Provision of products without medical examination	Purchase of food, medication refill, supplements
**Refusal of veterinary advice**	Veterinary recommendations were declined by the owner	Refusal of diagnostics, treatment, or hospitalization
**Symptomatic therapy including medication**	Treatment focused on relieving clinical signs	Pain relief, antiemetics, antibiotics
**Diagnostics**	Diagnostic procedures to identify or assess a condition	Blood tests, imaging, urinalysis, stool analysis
**Outpatient infusion therapy**	Fluid or infusion therapy without hospitalization	Subcutaneous or intravenous fluids during a visit
**Inpatient admission**	Hospitalization for ongoing monitoring or treatment	IV fluids, intensive care, postoperative monitoring
**Referral to veterinary hospital**	Transfer to a veterinary hospital or specialized facility or animal shelter with veterinary care	Referral for surgery, advanced imaging, intensive care, or shelter-based treatment
**Determination of death including euthanasia and resuscitation ^1^**	Confirmation of death or end-of-life procedures	Euthanasia, CPR attempts, death confirmation
**Wound care including dressing and surgery including anesthesia**	Treatment of wounds or surgical procedures	Bandaging, suturing, surgery under anesthesia, removing foreign bodies between the toes or awns from the paw
**Home visit**	Veterinary services provided at the owner’s home	Examination, euthanasia, follow-up care

Legend: CPR = cardiopulmonary resuscitation; IV fluids = intravenous fluids. ^1^ All animals included in this category were deceased by the end of the visit.

**Table 3:** Triage classification in dogs and cats presented after hours to a German private small-animal practice (*n* = 4579; 2021).

Triage	Urgency	Treatment Window	Classification	Description
**Red**	Immediate	Immediate	**True** **Emergency**	Visits requiring immediate veterinary assessment and treatment in an after-hours emergency setting
**Orange**	Very urgent	<10 min ^1^		
**Yellow**	Urgent	<30 min ^1^		
**Green**	Standard	<90 min ^1^	**Non-** **Emergency**	Visits not requiring urgent care and suitable for management during regular veterinary consultation hours
**Blue**	Non-urgent	<120 min ^1^		

^1^ min = minutes.

**Table 4:** Definition and distribution of age groups in dogs and cats presented after hours to a German private small-animal practice (n = 4579; 2021).

Age Group	Dogs	(*n* = 3189)	Cats	(*n* = 1390)
	Age (in Years)	%	Age (in Years)	%
Juvenile	0–1	25.2	0–1.5	25.1
Young Adult	1–6	41.9	1.5–6	22.5
Mature Adult ^1^	-	-	7–10	15.5
Senior	7–12	23.8	11–14	20.2
Old	13+	9.1	15+	16.6

^1^ Applicable for cats only.

**Table 5:** Most common descriptors of chief complaints within the presentation and medical care categories in dogs and cats presented after hours to a German private small-animal practice (*n* = 4579; 2021).

Reason for Presentation	*n*	%	Top 4 Descriptors (Highest to Lowest)			
No medical problem	178	3.9%	medication (*n* = 44)	tick (*n* = 34)	-	-
Gastrointestinal tract	1429	31.2%	vomiting (*n* = 739)	diarrhea (*n* = 478)	inappetence (*n* = 273)	abdomen (*n* = 162)
Respiratory tract	367	8.0%	cough (*n* = 192)	dyspnea (*n* = 83)	pulmonary edema (*n* = 25)	tachypnea (*n* = 25)
Poisoning	523	11.4%	induced vomiting (*n* = 375)	chocolate ingestion (*n* = 134)	foreign substance ingestion (*n* = 17)	-
Ear, nose and throat diseases (including eyes)	245	5.4%	cornea (*n* = 54)	awn ^1^ (*n* = 45)	conjunctivitis (n = 44)	-
Trauma and shock (including bleeding disorders)	382	8.3%	bite (*n* = 107)	high-rise syndrome (*n* = 69)	trauma (*n* = 38)	-
Kidney and urinary tract diseases	202	4.4%	cystitis (*n* = 81)	feline lower urinary tract disease (*n* = 38)	chronic kidney disease (*n* = 24)	-
Seizures	148	3.2%	epileptic seizure (*n* = 52)	seizure (*n* = 51)	vestibular (*n* = 23)	-
Musculoskeletal system (including lameness)	522	11.4%	lameness (*n* = 140)	pain (*n* = 114)	claw pathologies (*n* = 71)	-
Cardiovascular diseases	162	3.5%	cardiac (*n* = 33)	euthanasia (*n* = 31)	weakness (*n* = 18)	-
Metabolic diseases and allergies	304	6.6%	allergic (*n* = 91)	tick or insect bite (*n* = 58)	fever (*n* = 41)	-
Euthanasia	87	1.9%	home visit (*n* = 31)	tumor (*n* = 12)	-	-
Other	30	0.7%	pain (*n* = 19)	-	-	-
**Medical Care**	***n***	**%**	**Top 4 Descriptors (highest to lowest)**			
Diagnostics	1058	23.1%	X-ray (*n* = 377)	Blood test (*n* = 245)	ultrasound (*n* = 64)	blood pressure (*n* = 45)

^1^ hairy or bristle-like growth on a plant, e.g., a wild rye ear (spike).

## Data Availability

The data presented in this study are available from the corresponding author upon reasonable request, subject to applicable data protection and confidentiality requirements.

## Abbreviations

The following abbreviations are used in this manuscript:

GOT	Fee Schedule for Veterinarians [Gebührenordnung für Tierärzte]
HRS	High-rise syndrome
MTS	Manchester Triage System
VetTriS	Veterinary Triage System
VTL	Veterinary Triage List
